# A Systematic Review and Meta-Analysis on the Association and Differences between Aerobic Threshold and Point of Optimal Fat Oxidation

**DOI:** 10.3390/ijerph19116479

**Published:** 2022-05-26

**Authors:** Ratko Peric, Zoran Nikolovski, Marco Meucci, Philippe Tadger, Carlo Ferri Marini, Francisco José Amaro-Gahete

**Affiliations:** 1 Department for Exercise Physiology, Orthopedic Clinic Orthosport, 78000 Banja Luka, Bosnia and Herzegovina; 2 Faculty of Kinesiology, University of Split, 21000 Split, Croatia; zoranniko@yahoo.com; 3 Department of Health and Exercise Science, Appalachian State University, Boone, NC 28608, USA; meuccim@appstate.edu; 4 Real World Evidence, IQVIA, 3600 Genk, Belgium; philippetadger@gmail.com; 5 Department of Biomolecular Sciences, Division of Exercise and Health Sciences, University of Urbino Carlo Bo, 61029 Urbino, Italy; carlo.ferrimarini@uniurb.it; 6 Department of Physiology, Faculty of Medicine, University of Granada, 18001 Granada, Spain; 7 PROFITH “PROmoting FITness and Health through Physical Activity” Research Group, Department of Physical Education and Sport, Faculty of Sport Sciences, University of Granada, 18001 Granada, Spain

**Keywords:** AerT, exercise, FAT_max_, multilevel

## Abstract

Over the past two decades, scientists have attempted to evaluate whether the point of maximal fat oxidation (FAT_max_) and the aerobic threshold (AerT) are connected. The existence of such a relationship would allow a more tailored training approach for athletes while improving the efficacy of individualized exercise prescriptions when treating numerous health-related issues. However, studies have reported conflicting results, and this issue remains unresolved. This systematic review and meta-analysis aimed: (i) to examine the strength of the association between FAT_max_ and AerT by using the effect size (ES) of correlation coefficient (r) and standardized mean difference (SMD); (ii) to identify potential moderators and their influence on ES variability. This study was registered with PROSPERO (CRD42021239351) and ClinicalTrials (NCT03789045). PubMed and Google Scholar were searched and fourteen articles, consisting of overall 35 ES for *r* and 26 ES for SMD were included. Obtained ESs were analyzed using a multilevel random-effects meta-analysis. Our results support the presence of a significant association between FAT_max_ and AerT exercise intensities. In conclusion, due to the large ES variance caused by clinical and methodological differences among the studies, we recommend that future studies follow strict standardization of data collection and analysis of FAT_max_ and AerT-related outcomes.

## 1. Introduction

Lipids and carbohydrates are the dominant fuels utilized by humans during exercise with their absolute and relative contribution being influenced by sex, diet, exercise intensity and duration, time of the day, and fitness level [1]. During moderate exercise intensities, the energy contribution from lipids increases and then markedly declines to zero at heavy to severe exercise intensities; from that point on, carbohydrates become the dominant energy substrate [2,3]. Carbohydrates, due to their limited stores, can reduce performance during prolonged and/or heavy intensity activities; yet, as little as 1% of body fat can supply sufficient energy for up to 90 km of physical movement, making fat a more suitable fuel source [2]. Maximal fat oxidation point (FAT_max_) is commonly used to describe an exercise intensity at which fat oxidation is at its highest, whereas exercise intensity matching negligible fat oxidation is labeled FAT_min_ [3,4].

Regular exercise at FAT_max_ intensity has been proposed as a key factor to optimize the body’s ability to oxidize lipids, which is of the highest interest to athletes [5]. Moreover, with the current obesity epidemic representing a serious medical problem due to its association with numerous chronic diseases, (e.g., cardiovascular diseases, hypertension, diabetes), exercising at FAT_max_ intensity has also gained a great deal of attention among public health professionals and has been recommended for treating a number of chronic health issues [5,6,7]. Accurately prescribing exercise intensity is a complex task and there is controversy among both researchers and professionals regarding which of the methods used to design an efficient training plan is the most appropriate [8,9,10] The traditional approach is based on the prescription of exercise intensity as a percentage of maximal oxygen uptake (%VO_2max_) or maximal heart rate (%HR_max_), with these methods commonly represented in literature [8,9]. However, exercise intensity prescriptions based on %VO_2max_ have revealed moderate to large inter-subject variability (35–75%VO_2max_) at the intensities yielding FAT_max_ [1,8]. The variability of the FAT_max_ intensities becomes lower when %HR_max_ is used (55–65%HR_max_) yet remains ambiguous when it comes to individualized exercise prescription [11,12]. Hence, exercise intensities expressed as a fixed percentage of maximal values might not accurately reflect the metabolic responses of the human body [11,13,14]. For these reasons, some authors recommend that exercise intensity should be prescribed using a more standardized method, such as individual metabolic thresholds since traditional methods fail to account for differences in the subject’s metabolic stress [9,10,11]. In contrast to the relative percentage of VO_2max_ or HR_max_, an individualized approach to exercise intensity prescription based on metabolic thresholds describes specific metabolic phases during exercise and thus, intends to account for differences in the body’s physiological and functional capacity [11]. This approach might also homogenize the elicited metabolic stress and consequently reduce individual variability in metabolic responses despite differences in their phenotype [9,15].

During exercise with increasing intensity, three phases of the body’s energy production and two threshold points delineating these phases can be distinguished [15]. These threshold points have been termed the metabolic thresholds and can be determined by either gas analysis or blood lactate techniques [15,16]. Throughout the years, scientists used different terms to identify these two thresholds, whether they wanted to refer to the physiological processes occurring in the body or to the methods used to identify them [11,15,16]. For additional clarification of the physiological and methodological significance of the thresholds, we suggest further reading [11,15,16]. In this paper, we will mention only the first threshold, whereas the term aerobic threshold (AerT) will be used to refer to it. Our goal is to align with the conceptual framework for performance diagnosis and training prescription proposed and clearly described by Meyer et al. (2005) [15].

Ever since the term FAT_max_ was introduced, scientists have tried to determine the existence of a relationship between exercise intensities matching FAT_max_ and AerT, with the aim of assuring a more individualized exercise prescription [17,18,19]. If such a connection exists, it would integrate the most relevant indices for planning and assessing an effective exercise program [9,10]. Hence, over the last two decades, conflicting results with high inter-study variability on the association between exercise intensities matching FAT_max_ and AerT have been reported [20,21,22]. These variations may have resulted from both methodological and clinical differences within and between the studies [12]. To our knowledge, no studies have systematically explained this variability.

Hence, this systematic review and meta-analysis aimed to examine the association between the FAT_max_ and AerT, identify relevant moderators, and examine their influence on effect size variability.

## 2. Materials and Methods

### 2.1. Study Design

This systematic review was registered in the International Prospective Register of Systematic Reviews (PROSPERO) (ID: CRD42021239351) and is part of a pre-registered trial on ClinicalTrials.gov (ID: NCT03789045). The Preferred Reporting Items for Systematic Reviews and Meta-Analyses (PRISMA) checklist and flow diagram for reporting systematic reviews and meta-analyses was used as a tool to structure this review and describe the methodology, and systematically present our search findings [23].

### 2.2. Search Strategy

We searched MEDLINE (via PubMed) and Google Scholar for studies exploring the FAT_max_ and AerT relationship. The search was performed using the Boolean logic, which limits the search results with operators including AND/OR to only those documents containing relevant key terms in the scope of the review. The search combined the following key terms: “fat oxidation”, OR “maximal fat oxidation”, OR “optimal fat oxidation”, OR “peak fat oxidation”, AND “aerobic threshold”, OR “anaerobic threshold”, OR “ventilatory threshold”, OR “lactate threshold”, OR “metabolic threshold”, OR “gas exchange threshold”, representing variety in terminology used [15,16]. The search was developed and conducted by two independent researchers (PR and NZ) and reported using the PRISMA statement [23]. Furthermore, since the FAT_max_ phrase was introduced in 2001 [2], the inclusion date for the publications was restricted from 1 January 2001, to present. The last search was run on 1 April 2021.

### 2.3. Data Extraction

Two investigators (PR and NZ) independently performed, in an unblinded standardized manner, screening of the retrieved records, and checked whether they met the eligibility criteria, with a third reviewer being involved when opinion differences were present (FMC). Data were extracted from each of the selected articles and summarized in an Excel spreadsheet (Microsoft, Redmond, WA, USA) for further analysis.

### 2.4. Inclusion/Exclusion Criteria

Research articles were selected using the defined population, intervention, comparison, outcomes, and study (PICOS) design [24], with study characteristics for inclusion established as: (i) original studies, (i.e., randomized and non-randomized controlled trials, cohort studies, case–control studies, and cross-sectional studies) in form of (ii) full text, abstracts or congress presentations. Furthermore, the studies included had to report AerT and FAT_max_ intensities occurrence, (i.e., mean ± SD) and their association, (i.e., Pearson correlation, Kendall rank correlation, or Spearman correlation coefficient). If one of two requirements failed to be reported or was unable to be determined, the corresponding author was contacted. However, independent of its form, to be considered eligible, these studies were required to show a clear and reproducible description of the methods used to determine both AerT and FAT_max_. Case reports, editorials, reviews, and opinion papers were excluded. In addition, only studies that included participants with no evidence of any metabolic, pulmonary, or cardiovascular diseases (conditions potentially affecting substrate utilization) were considered acceptable. No other restrictions to participants’ characteristics were introduced other than age (18–60 years). Report criteria required studies to be written in English and to be published in a peer-reviewed journal. The grading of recommendations, assessment, development, and evaluation (GRADE) approach was applied to define the quality of a body of evidence for selected studies [25].

### 2.5. Statistical Analysis

All statistical analyses were performed with R software (version 4.0.4) (The R Foundation, Vienna, Austria) by using *metafor* and *dmetar* packages [26,27]. Pearson’s correlation coefficients (*r*) and standardized mean difference (SMD) between the exercise intensities at which AerT and FAT_max_ appeared were used as ESs; such ESs were analyzed separately to estimate the strength of the correlation and the size difference between the two exercise intensities, respectively. Summary SMD and *r* estimates were determined using a random-effects model and presented as mean and 95% confidence (CI) and prediction (PI) interval [28].

Considering that *r* can be biased by the measurement unit used to compute it due to possible spurious correlation, a meta-analysis of *r* had to be performed separately for each measurement unit used to identify exercise intensity at FAT_max_ and AerT [29]. Indeed, the correlation between variables that are non-independent due to the share of a common denominator, (i.e., variables expressed as a fraction of body weight or maximal values), can be influenced and over-estimate the correlation between the original independent variables [29,30]. On the other hand, SMD allows ESs deriving from different measurement units to be pooled in one single meta-analysis [30].

To account for dependencies among ESs, which in certain cases were clustered within the same study, multilevel meta-analyses (MA) were performed where the level 1 variance is attributed to pure sampling error, the level 2 described the within-studies variance, and level 3 represented the amount of between-studies variance [31,32,33]. Distribution of variance among the different levels was used to identify the required levels of modeling [33]. Moreover, the model assumptions about the identifiability of each parameter (the variances of each model) were checked using the profile likelihood (PL) of each fitted model; when the PL did not provide an identifiable profile of the parameter, a simpler model was considered [34]. Sensitivity analysis based on the leave-one-out method and statistical outlier detection test was implemented in order to explore the impact of excluding or including any ES, determine the impact of distortion on the pooled overall effect estimate as well as avoid potential pseudoreplication problems [35,36]. In a multilevel meta-analysis, the risk of bias across studies (publication bias) requires a specialized analysis; thus, publication bias was determined by an extended Funnel plot test and the adapted Funnel plot for multilevel meta-analysis [37]. The risk of bias in individual studies, (i.e., internal validity) included in this paper was examined using a ROBIS tool, with results of this assessment presented in a graphical format [38].

For each outcome parameter, degrees of heterogeneity were measured with Cochran’s test for chi-squared statistic of total (Q) and expected variance (df) and expressed as the Higgins (I^2^) statistic [39,40]. If heterogeneity was present, the random-effects model was the preferred model, and the weighing factor, the inverse of the between-studies and within-studies variance was used [28]. Additionally, the PI calculated for each ES provided a predicted range for the true treatment effect in an individual study, additionally describing the degrees of heterogeneity between the studies [26]. In cases of relevant heterogeneity between the studies (0% to 40%: might not be important; 30% to 60%: may represent moderate heterogeneity; 50% to 90%: may represent substantial heterogeneity; 75% to 100%: considerable heterogeneity), relevant moderators and matching subgroups were identified and an analysis of changes in variation in effects was performed [33,41]. For each SMD and *r* meta-analysis, potential sources of heterogeneity in a test of moderators were assessed by using an omnibus test based on F distribution [42] which was performed solely if at least three ESs were available per subgroup [33]. The standardized measure of effect size (Cohen’s F) was also used to describe the strength and practical significance of the test of moderator, with values *f* = 0.1 is a small effect, *f* = 0.25 is a medium effect, and *f* = 0.4 is a large effect, respectively [43]. The critical value for the F distribution (*f^2^*) was determined from the table with the α = 0.05 [43].

When *r* was used as the ES, if measures of association other than Pearson’s correlation (*r*) were reported, they were converted to *r* as previously described [44]. Moreover, to obtain unbiased weights for each study, Fisher’s *z*-transformation was used to convert *r* values derived from the original studies to *z* values, which were used for the statistical analyses (*z* values were transformed back to *r* for presentation purposes) [28,45]. Correlations were classified as weak (𝑟 ≤ 0.30), moderate (𝑟 = 0.30–0.50), significant (𝑟 = 0.50–0.70), and strong (𝑟 > 0.70) [46].

When SMD was used as the ES, means and SD values of exercise intensities at FAT_max_ and AerT were extracted from all the retrieved studies. The SMD was computed to account for the differences between FAT_max_ and AerT since the studies assessed the same outcome, (i.e., exercise intensity at FAT_max_ and AerT) but measured them by using different measurement units [47]. Additionally, since FAT_max_ and AerT exercise intensities were paired data deriving from the same individual, the SMD was computed to account for paired measures by accounting for the correlation between the two exercise intensities [48]. Treating the two intensities as independent measures could rise the unit-of-analysis error by providing confidence intervals that are likely to be too wide, and reducing the trial’s weight, with the possible consequence of disguising clinically important heterogeneity [39]. For one study where *r* was not reported [49], *r* had to be calculated using a different measurement unit than that one used to report mean and SD values, (i.e., %VO_2max_) and therefore used to compute SMD which was assumed to be equal to the pooled *r* derived from the same measurement unit as ES. Cohen’s rule of thumb for interpretation of the SMD statistic was followed: a value of ≤0.2 indicates no effect, a value of 0.21 to 0.5 indicates a small effect, a value of 0.51 to 0.8 indicates a medium effect and a value of >0.81 indicates a large effect [43].

A descriptive exploration of the plotted ESs (*r* and SMD) was used as a proxy of agreement, where high agreement was assumed in case of high *r* and small effect SMD (*r* > 0.7 and SMD < 0.5) and low agreement in case of low *r* and medium to large effect SMD (*r* < 0.7 or SMD > 0.51). Furthermore, this method was used to explore measurement units as a potential source of spuriousness [29].

## 3. Results

### 3.1. Descriptive Results

**Figure 1:**
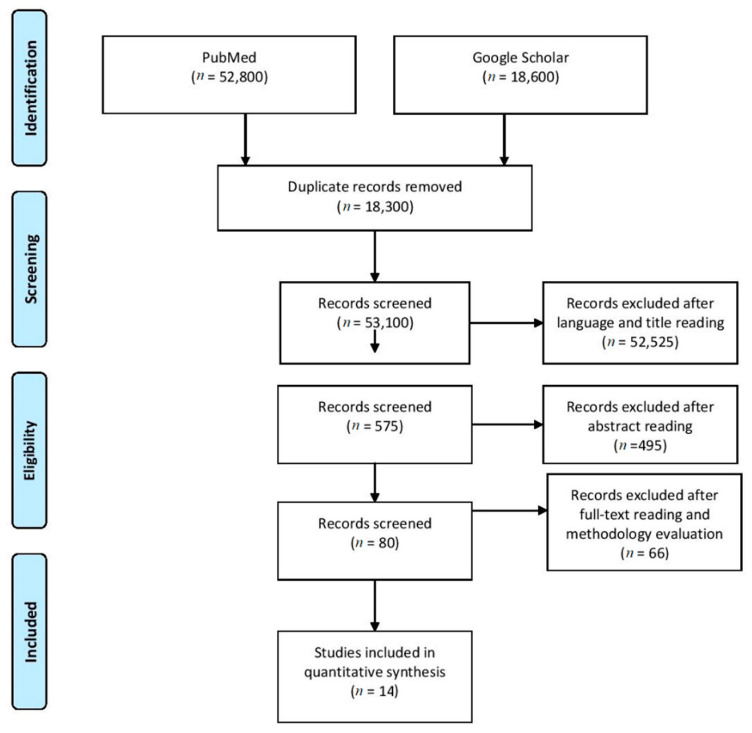
PRISMA flow diagram of the literature search and study selection process.

**Figure 2:**
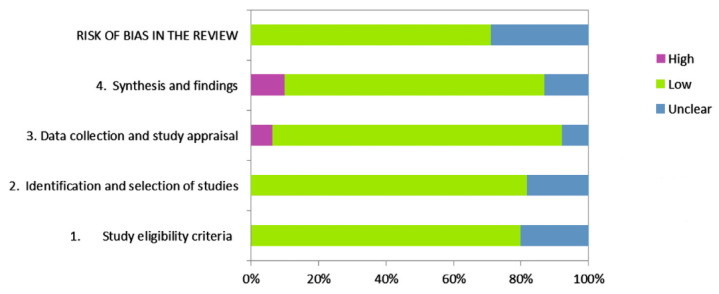
Graphical presentation for ROBIS results.

The systematic search retrieved a total of 71,400 papers with 18,300 identified as duplicates. Thereafter, screening of the paper’s title and language was performed with a total of 575 potentially relevant papers included in the abstract screening. Subsequently, eighty records were selected to be read in full as potentially eligible. An additional sixty-six articles were excluded after reading the full text as they did not fulfill the inclusion criteria. Data extraction was performed by two reviewers (PR and NZ) independently, using a data extraction Excel form (Microsoft, Redmond, WA, USA). Any disagreements about data extraction were solved by consensus or by the decision of a third reviewer. For one study, correlation coefficients were converted from Kendall’s tau to Pearson *r* [20]. The literature search and study selection process are reported using the PRISMA statement in Figure 1. The internal validity of included studies is presented in Figure 2. One correlation ES was excluded as it was identified as an outlier after sensitivity analysis (results not shown) due to its ES estimate being too extreme to fit within pooled ES and not overlapping with the 95% CI of the pooled effect [50]. Moreover, its ES overly contributed to the heterogeneity, while at the same time was not very influential concerning the overall pooled weight (presumably due to the small sample size). Overall, fourteen papers were included in the present study with a total of 35 ES for *r* and 26 ES for SMD.

### 3.2. Exploring Heterogeneity and Subgroup Analysis

Due to anticipated heterogeneity, three investigators (PR, AGFJ, and FMC) independently performed the identification of common variables, (i.e., relevant moderators) and their subgroups allowing sources of variation to be investigated. Identified moderator variables, categorized by differences in characteristics of the studies, (i.e., methodological diversity) and by study populations, (i.e., clinical diversity), are reported in Table 1.

### 3.3. Clinical Diversity

When the selected studies were examined for their clinical diversity, two moderators were identified: (i) sex and (ii) physical activity level, each with two subgroups (Table 1). All included papers collectively evaluated 855 participants, out of which 526 (61.52%) were males and 329 (38.48%) were females. Seven studies (50%) used males as primary participants, whereas one (7.14%) study tested only females. In six (42.86%) studies, both sexes were evaluated. When classified by physical activity level, 296 (34.62%) subjects were active while 559 (65.38%) were inactive, (i.e., sedentary or obese). Seven studies (50%) examined the active population whereas six (42.86%) studies examined inactive participants. Only one study (7.14%) examined both groups.

### 3.4. Methodological Diversity

When selected studies were examined for their methodological diversity, six moderators with relevant subgroups were identified (Table 1). Cycle ergometry was the preferred method in nine (64.29%) studies, whereas treadmill was employed in five (35.71%). When considering the methods used to determine the AerT, five (35.71%) studies preferred the lactate method whereas gas analysis was used in seven (50%). Two (14.29%) times, both methods were used. When evaluating the measurement methods used to establish correlation, five methods were identified: thirteen (37.14%) cases preferred mL/min/kg with L/min, %VO_2max_ and b/min were used in seven (20%), and %HR_max_ in two (5.71%) cases. The analysis of test protocols used to assess VO_2max_/AerT, showed that all studies favored a graded exercise test (GXT) type protocol. Twelve (85.71%) studies preferred shorter stages (≤3-min) while longer stages (>3-min) were used in only one study (7.14%). One (7.14%) study examined the association by using both short and long stages. FAT_max_ was identified by using visual inspection of the appropriate plots in nine (64.29%) studies while five (35.71%) studies approached this issue by using a mathematical model. Finally, ten (71.43%) studies determined FAT_max_ during the same GXT used to assess VO_2max_/AerT, whereas four (28.57%) studies used an additional test.

### 3.5. Meta-Analysis Study of Correlation Coefficients

#### 3.5.1. Overall Effect

All data provided by the selected studies met the inclusion standard for correlation meta-analysis with the studies evaluated for their heterogeneity and bias. Pooled ESs calculated using random effects, 95% CI, and PI with the results of heterogeneity and distribution of variance results for each measurement unit are presented in Table 2. By observing the adapted Funnel plot for multilevel meta-analysis (results not shown) for each measurement unit used to assess r, we observed that the distribution of study-specific effects was actually quite symmetrical, with no indication of the existence of publication bias.

#### 3.5.2. B/min Measurement Method

For the observed measurement method, only one moderator met the criteria set to perform the test of moderators.

##### Sex

In the observed data (with the sub-grouping) the PL for the three-level MA was non- informative, so a two-level MA was fitted. When sex was examined as a moderator, the test of moderators revealed no statistical differences (F_1,4_ = 0.54, critical *f*^2^ = 7.71, *p* = 0.502). For this moderator, pooled values for a common estimate of heterogeneity showed Q = 46.57, df = 4, I^2^ = 91.19, *p* < 0.001. For males, mean ES was 0.50 (95% CI −0.03 to 0.81; PI −0.35 to 0.90) whereas for females, mean ES was 0.71 (95% CI −0.75 to 0.99; PI −0.99 to 1.00). Total heterogeneity for females was Q = 42.42, df = 2, I^2^ = 94.56%, *p* < 0.001. For males, heterogeneity was Q = 4.16, df = 2, I^2^ = 52.14%, *p* < 0.125.

#### 3.5.3. mL/kg/min Measurement Method

For the observed measurement method, the following moderators met the inclusion criteria set to perform the test of moderators.

##### Sex

In the observed data (with the sub-grouping) the PL for the three-level MA was non-informative, so a two-level MA was fitted. When sex was examined as a moderator, no statistical differences were noticed (F_1,11_ = 0.01, critical *f*^2^ = 4.84, *p* = 0.994). For this moderator, pooled values for a common estimate of heterogeneity showed Q = 44.70, df = 11, I^2^ = 75.87, *p* < 0.001. For males, mean ES was 0.73 (95% CI 0.50 to 0.86; PI −0.03 to 0.96) whereas for females, mean ES was 0.74 (95% CI 0.56 to 0.85; PI 0.29 to 0.92). Total heterogeneity for females was Q = 12.22, df = 5, I^2^ = 59.71%, *p* < 0.032. For males, heterogeneity was Q = 32.47, df = 6, I^2^ = 81.95%, *p* < 0.001, respectively.

##### Physical Activity

For this moderator, the PL for the three-level MA was informative, so this was the fitted model. Test of moderators (F_1,11_ = 0.03, critical *f*^2^ = 4.84, *p* = 0.874) concluded that both subgroups had similar variances. Pooled heterogeneity was high (Q = 40.12, df = 11, I^2^ = 78.93%, *p* < 0.001). The analysis of this moderator and its subgroups showed mean ES for active (0.77, 95% CI 0.40 to 0.92; PI −0.11 to 0.97) and inactive (0.79, 95% CI 0.58 to 0.90; PI 0.25 to 0.95) individuals to be similar. Substantial I^2^ (93.16%) for the active group (Q = 26.32, df = 5, *p* < 0.001) originated mostly from level 3 (79.31%) and was negligible from level 2 (3.85%). For inactive individuals, heterogeneity (Q = 13.81, df = 6, *p* < 0.032, I^2^ = 71.5%) derived negligible from level 2 (5.43%) and considerably from level 3 (66.07%).

##### Ergometer

The PL for the three-level MA was informative, so this was a fitted model. When the type of ergometer was examined for existence of variance, no statistical differences were revealed (F_1,11_ = 0.47, critical *f*^2^ = 4.84, *p* = 0.053). For this moderator, the presence of heterogeneity was (Q = 29.87, df = 11, *p* < 0.002) divided between level 2 (7.45%) and level 3 (55.59%), respectively. For the treadmill, mean ES was 0.86 (95% CI 0.71 to 0.93; PI 0.72 to 0.93) whereas for the cycle ergometer, mean ES was 0.69 (95% CI 0.48 to 0.83; PI 0.15 to 0.92). Identified heterogeneity for the treadmill was (Q = 1.54, df = 2, I^2^ = 0.00%, *p* < 0.462). For cycling, observed heterogeneity was (Q = 28.33, df = 9, *p* < 0.001) and originated from level 3 (63.38%) and minimally from level 2 (7%).

##### AerT Detection Method

The PL for the three-level MA was informative, so this was the fitted model. Test for subgroup differences showed F_1,11_ = 1.55, critical *f*^2^ = 4.84, *p* = 0.240. Pooled ES for gas analysis was 0.82 (95% CI 0.66 to 0.90; PI 0.41 to 0.95) and for lactate method 0.67 (95% CI 0.33 to 0.86; PI −0.07 to 0.94). With an overall high heterogeneity identified (Q = 11.18, df = 11, *p* = 0.001) divided between level 2 (6.22%) and level 3 (65.21%), moderate heterogeneity (I^2^ = 58.14%) was observed in cases using gas analysis, whereas high heterogeneity (I^2^ = 79.12%), unevenly distributed between level 2 (8.57%) and level 3 (70.55%) in cases using the lactate method was detected.

##### FAT_max_ Test

For this moderator, the PL for the three-level MA was informative, so this was the fitted model. When the test used to detect FAT_max_ was analysed, two subgroups were identified: (i) FAT_max_ determined during VO_2max_/AerT test, (ii) FAT_max_ determined with an additional test (F_1,11_ = 2.38, critical *f*^2^ = 4.84, *p* = 0.151). For this moderator, pooled values for a common estimate reported substantial heterogeneity (Q = 40.05, df = 11, I^2^ = 71.44% (level 2 = 5.77% and level 3 = 65.67%), *p* < 0.001). For the first subgroup, mean ES was 0.80 (95% CI 0.68 to 0.88 and PI 0.40 to 0.95) whereas for additional test group, mean ES was 0.53 (95% CI −0.15 to 0.87 and PI −0.15 to 0.87). Even though total I^2^ for the identical VO_2max_ protocol subgroup was moderate (Q = 39.20, df = 9, I^2^ = 75.54%, *p* < 0.001), it originated mainly from level 3 (67.47%) with small contribution from level 2 (8.08%). In the case of the additional test, no heterogeneity was noted (Q = 0.00, df = 2, I^2^ = 0.00%, *p* < 0.653).

#### 3.5.4. %O_2max_ Measurement Method

For the observed measurement method, the following moderators met the inclusion criteria set to perform the test of moderators.

##### Physical Activity

For this moderator, the PL for the three-level MA was non-informative, so the fitted model was a two-level. Results of test of moderators were F_1,5_ = 0.01, critical *f*^2^ = 6.61, *p* = 0.982. Pooled heterogeneity was Q = 74.79, df = 6, I^2^ = 93.93%, *p* < 0.001. Subgroups analysis showed mean ESs for active (0.55, 95% CI −0.83 to 0.96; PI −0.99 to 1.00) and inactive (0.55, 95% CI −0.19 to 0.89; PI −0.81 to 0.98) individuals to be similar. Substantial I2 (85.19%) was observed for the both active (Q = 16.34, df = 2, *p* < 0.001) and inactive individuals (Q = 33.07, df = 3, *p* < 0.001, I^2^ = 94.47%), respectively.

##### Ergometer

The PL for the three-level MA was non-informative, so the fitted model was a two-level. Results for the test of moderators were F_1,5_ = 0.76, critical *f*^2^ = 6.61, *p* = 0.793. For this moderator, the presence of heterogeneity was high (Q = 74.45, df = 5, I^2^ = 93.81%, *p* < 0.001). For the treadmill, mean ES was 0.59 (95% CI −0.21 to 0.92; PI −0.86 to 0.99) whereas for the cycle ergometer, mean ES was 0.50 (95% CI −0.79 to 0.98; PI −0.98 to 1.00). Total identified heterogeneity for the treadmill was (Q = 67.21, df = 3, I^2^ = 96.42%, *p* < 0.001). For cycling, observed heterogeneity was Q = 7.44, df = 2, I^2^ = 72%, *p* < 0.001.

##### FAT_max_ Detection Method

The PL for the three-level MA was non- informative, so a two-level MA was fitted. When evaluating the FAT_max_ detection method as a moderator, two subgroups were identified: visual and mathematical methods (F_1,5_ = 0.06, critical *f*^2^ = 6.61, *p* = 0.823). For the visual method of detection, mean ES was 0.52 (95% CI −0.76 to 0.97 and PI −0.99 to 1.00) whereas for the mathematical method, mean ES was 0.60 (95% CI −0.18 to 0.92 and PI −0.77 to 0.98). For this moderator, pooled values for a common estimate confirmed the existence of substantial heterogeneity (Q = 60.71, df = 5, I^2^ = 93.86%, *p* < 0.001). Total I^2^ for the visual inspection method was considerably high (Q = 50.48, df = 2, I^2^ = 97.61%, *p* < 0.001). For the mathematical method, heterogeneity was substantial (Q = 10.22, df = 3, I^2^ = 73.40%, *p* < 0.001). The expected effect for future studies in both groups varied from very weak to very strong, reflecting high heterogeneity.

#### 3.5.5. L/min Measurement Method

For the observed measurement method, no moderators met the inclusion criteria set to perform the test of moderators.

### 3.6. Meta-Analysis Study of Standardised Mean Differences

#### 3.6.1. Overall Effect

**Figure 3:**
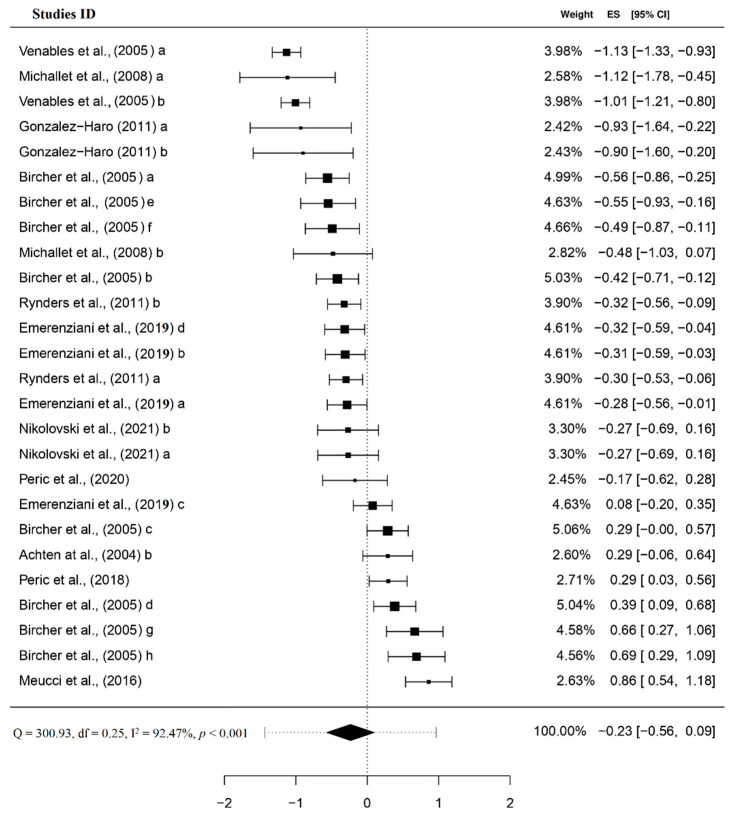
Forest plot reporting SMD. *NOTE:* Reported values for each study are presented by using random-effect model weights, ES, and 95% CI [12,18,19,20,21,22,49,52,53,54,55].

Since SMD allows adjustment for different effect sizes deriving from different measurement units without bias presence, calculated SMDs were pooled in one single meta-analysis. ESs were calculated using random effects, and 95% CI with the results of heterogeneity, and the distribution of variance results for each measurement unit is presented in Figure 3. Polled PI was −1.43 to 0.97 with SE = 0.16. Residual heterogeneity was substantial and similarly divided between level 2 (39.75%) and level 3 (52.73%). In 69.23%, FAT_max_ preceded AerT whereas the test of moderators revealed that only in the case of long stage GXT did AerT tend to precede FAT_max_ (Table 3). By observing the adapted funnel plot for multilevel meta-analysis (results not shown) for each measurement unit used to assess SMD, we observed that the distribution of study-specific effects was actually quite symmetrical, with no indication of the existence of publication bias.

#### 3.6.2. Sex

For this moderator, the PL for the three-level MA was informative, so this was the fitted model. Test of moderators revealed F_1,22_ = 0.01, critical *f*^2^ = 4.30, *p* = 0.936. For this moderator, pooled values for a common estimate of heterogeneity showed Q = 290.54, df = 22, *p* < 0.001 with variance equally being distributed between level 2 (41.65%) and level 3 (51.49%).

#### 3.6.3. Physical Activity

For this moderator, the PL for the three-level MA was informative, so this was the fitted model. Test of moderators results were F_1,24_ = 1.20, critical *f*^2^ = 4.260, *p* = 0.285. Residual heterogeneity (Q = 223.48, df = 24, *p* < 0.001) variance was distributed across level 2 (39.24%) and level 3 (53.11%).

#### 3.6.4. Ergometer

The PL for the three-level MA was informative, so a three-level MA was used. When the type of ergometer was examined, the results were F_1,24_ = 0.33, critical *f*^2^ = 4.260, *p* = 0.572. For this moderator, the presence of residual heterogeneity was confirmed (Q = 285.26, df = 24, *p* < 0.001) with I^2^ originating from level 2 (35.06%) and level 3 (58.09%).

#### 3.6.5. VO_2max_/AerT Test

The PL for the three-level MA was non-informative for the VO_2max_/AerT test subgroups, so a two-level MA was fitted. The test for subgroup differences showed F_1,24_ = 3.19, critical *f*^2^ = 4.260, *p* = 0.087 with substantial residual heterogeneity present (Q = 237.21, df = 24, I^2^ = 89.9%, *p* = 0.001).

#### 3.6.6. AerT Detection Method

The PL for the three-level MA was informative, so this was the fitted model. Tests for subgroup differences in AerT detection methods showed F_1,24_ = 0.03, critical *f*^2^ = 4.260, *p* = 0.857. With an overall high heterogeneity identified (Q = 281.50, df = 24, *p* < 0.001), substantial heterogeneity (I^2^ = 95.23%) was distributed within (1.3%) and between (93.93%) studies using gas analysis, whereas moderate heterogeneity (Q = 92.03, df = 13, I^2^ = 89.41%, *p* < 0.001), unevenly distributed between level 2 (71.62%) and level 3 (17.79%) in studies using the lactate method were detected.

#### 3.6.7. FAT_max_ Detection Method

The PL for the three-level MA was informative. When evaluating the FAT_max_ detection method, two subgroups were identified: visual and mathematical methods, with following results (F_1,24_ = 1.76, critical *f*^2^ = 4.260, *p* = 0.197). For this moderator, pooled values for a common estimate confirmed the existence of substantial heterogeneity (Q = 300.77, df = 24, I^2^ = 92.39% (level 2 38.67% and level 3 53.72%), *p* < 0.001). The expected effect for future studies in both groups varied from very weak to very strong, reflecting high heterogeneity.

### 3.7. Correlation Coefficients and Standardized Mean Differences

**Figure 4:**
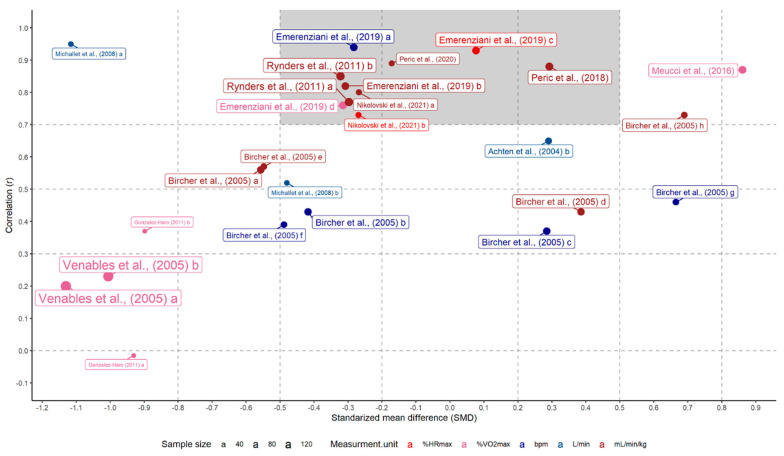
Correlation coefficients vs. SMD plot for exercise intensities methods, categorized by measurement units. *NOTE*: Bluish colors are used for display of potentially non-spurious measurement units, (i.e., bpm and L/min), and the reddish colors are used for display of potentially spurious measurement units, (i.e., %HR_max_, %VO_2max_, and mL/min/kg), respectively [12,18,19,20,21,22,49,52,53,54,55].

Finally, r vs. SMD between the exercise intensities at FAT_max_ and AerT were plotted in Figure 4; providing an integrated view of all ESs. The combination of *r* and SMD can be seen as a proxy of agreement, where high agreement is observed in the case of high *r* and low SMD (*r* > 0.7 and SMD < 0.5, represented by the dark grey square in Figure 4) and low agreement related to low r or high SMD (*r* < 0.7 and SMD > 0.51, white background in Figure 4).

## 4. Discussion

The main conclusion of our study was that the FAT_max_ and AerT connection exhibits a consistent and strong association. Moreover, the variations in methodological designs of the studies, combined with the clinical diversity seem to contribute to the apparently inconsistent, (i.e., heterogeneous) results. These findings represent an important practical application that allows a more tailored and individualized approach to exercise prescription.

The Strongest *r*, with the narrowest 95% CI and PI, was observed in the case of mL/min/kg, implying the highest association when this method is used. Test of moderators followed by the subgroups analysis revealed no influence of clinical differences on FAT_max_ and AerT correlation. However, substantial heterogeneity was still noticed for both clinical moderators which remained considerable even after subgroup analysis. If heterogeneity is lower within the subgroups than across the pooled data, we can assume that the subgroup analysis contributes toward explaining heterogeneity in the overall analysis. However, this was not the case. Potential explanations could be drawn from the characteristics of study participants, (i.e., demographics, sex, age range, body composition, etc.), which in our study could not be controlled. Furthermore, stronger correlation and narrower 95% CI and PI were noticed in the studies using a treadmill, gas analysis for AerT detection, and a single GXT used to determine AerT and FAT_max_, respectively. These findings suggest more robust and reliable results since CIs can be interpreted as an estimate of the “true” effect. A sufficient number of participants were included in each subgroup, so the covariate distribution is not concerning in these results. As a general rule, the larger the differences between the subgroups and narrower their CI’s, the more robust and reliable the results are. Concerning methods used to detect AerT, results for the lactate subgroup indicate large dispersion in the correlations; hence, the results observed cannot be considered deriving from a single population. Contrary, gas analysis was revealed to be a more reliable method when comparing exercise intensities of AerT and FAT_max_, with the lactate method significantly reducing the strength of the association. However, it is worth noting that the observed presence of considerable heterogeneity for each subgroup could originate from multiple methods associated with either gas analysis or lactate tests, which require further examination [56]. Based on the available data, it is recommended that future trials should prefer gas analysis over lactate methods when aiming to assess the correlation between AerT and FAT_max_. When considering the test used to determine FAT_max_, no heterogeneity was identified for an additional test, presumably due to the small number of included studies. However, in the group where FAT_max_ and AerT were determined during a single GXT, substantial heterogeneity was revealed, presumably as a result of methodological differences among the studies. These differences can probably be attributed to different GXT protocol methodologies (stages length and incline differences), contributing toward subgroup variability. In our MA, stages ranging from 1 min to 6 min were reported (Table 1). However, due to the small number of included studies and to adequately assess statistical significance, stages ≤3 min were classified as short whereas, stages >3 min were classified as long [13,14]. Even though reliability was higher for the short stages group, it still remains vague if 1-min, 2-min or 3-min stages are more adequate as no consensus exists regarding the optimal GXT methodology when determining VO_2max_/AerT [1,8,13]. Hence, using a single GXT should be a preferable method, which, in the long run, would allow further insight into the variability within the short stages. Furthermore, this strategy could inhibit potential pseudoreplication problems, while at the same time being less time-consuming and more economical for both researchers and subjects [12,36].

Even though the L/min measurement method was revealed to have the second strongest correlation coefficient, no moderators met the inclusion criteria set to perform the test of moderators. However, this method revealed robust and reliable results due to narrow 95% CI and associated PI, demonstrating a possible future application of this method. However, since a non-sufficient number of participants were included in each subgroup, the covariate distribution is a concerning factor for this analysis.

When the b/min measurement method was used, lower *r* with a wider 95% CI and PI were observed, compared to previously discussed methods, with only one moderator meeting the required standards for the test of moderators. Nevertheless, sex was revealed to have no influence on the correlation strength with a somewhat stronger correlation and wider 95% CI and PI observed in females compared to males. This result could be attributed to the substantially larger number of males included in this test of moderators, allowing more robust results. Considering this, future studies should expect a high variability in their results when this method of correlation assessment is used.

Even though the %VO_2max_ was observed as the method with the weakest correlation, none of the tested moderators were revealed to have a significant influence on the correlation strength. This result, in line with our original framework, goes to show that the individual metabolic responses to exercise cannot accurately be identified by calculating fixed percentages of maximal values [11,13,14]. Moreover, future studies using this method could expect large heterogeneity in their results since 95% CI and PI observed were wide, leading to low reliability of obtained results since obtained CIs cannot be interpreted as an estimate of the “true” effect.

The SMD analysis showed a small negative pooled effect size, revealing no significant differences between the intensities at which FAT_max_ and AerT occur, with a strong tendency of FAT_max_ to precede AerT. Furthermore, it seems that the duration of the stages used during an exercise test for VO_2max_/AerT determination may affect the association between FAT_max_ and the AerT. When long stages are used in a GXT, AerT tended to precede FAT_max_, demonstrating a direct influence of the specific moderator on the order. The fact that longer stages tend to underestimate FAT_max_ could be a potential reason for this occurrence [1,12]. This finding advises—when prescribing individualized exercise—researchers and professionals should devote their attention toward methods standardization considering the VO_2max_/AerT identification as that will also optimize the oxidation of fat [9,10,11]. The heterogeneity for the pooled SMD meta-analysis was high, with a tendency of lowering during the subgroup SMDs analysis; indicating an influence of the methodological differences on the exercise intensities matching FAT_max_ and AerT. The test of moderators showed no significant effect for any of the moderators considered, which, as for the *r* ES analyses, seems to highlight that most of the heterogeneity was due to multiple factors caused by the lack of standardized methods and guidelines to assess the association between FAT_max_ and AerT.

When *r* and SMD ESs were plotted (see Figure 4), no evident pattern in relation to the moderators tested was noticed. Out of the 25 matched correlation coefficients and SMDs, 10 showed high *r* and low SMD, which seemed to be relatively equally distributed between the moderators fulfilling the requirement for the test of moderators. Moreover, although high *r* and low SMD Ess seemed to be more frequent in certain subgroups, (i.e., short stages VO_2max_ protocol, single GXT, and gas analysis method), there are several other matched ESs computed from studies that used mentioned moderators, yet not reported high *r* and low SMD. Interestingly, when the studies reporting high *r* and low SMD were considered, there seemed to be a pattern. In fact, if a matched pair of ESs showed high *r* and low SMD, this was also true for all the other ESs of that specific study. This implies that the combination of low SMD and high *r* was relatively uniform within the study, suggesting the existence of possible further methodological or clinical differences that remained uncovered during the moderators’ analysis. Studies with measurement units such as b/min or L/min, which can be considered as non-spurious measures (not normalized to subjects), show a tendency to have weak to moderate correlation values or high SMDs; with one study as the only exception [18]. Contrary, the studies with low SMD and high correlation values tend to be studies that use a potentially spurious measures such as %HR_max_, %V0_2max_, and mL/min/kg (see the dark grey rectangle in Figure 4). This may be due to the fact that when the exercise intensity is expressed in relative terms, the interindividual variability is reduced as the exercise intensity is normalized and uniform among individuals.

The present study is not without limitations. First, this systematic review included only English written peer-reviewed published studies restricted to healthy adult subjects pooling data from nonrandomized studies. Therefore, many confounding factors that might affect the correlation between FAT_max_ and AerT could not be controlled. Second, although the number of participants included in the study was large, numbers in the several subgroups were relatively limited, inhibiting appropriate statistical analysis. Third, although these findings strongly suggest that a relationship exists between the two intensities, correlations and SMD are not sufficient to confirm that the intensities necessarily coincide and that the error between the two measures is small. Indeed, agreement and correlation are widely-used concepts that assess the relationship between variables and although similar and related, they represent different notions of connection [57]. Due to the lack of the information needed to directly assess agreement in the selected studies, our results could not provide direct information regarding the associated levels of agreement and error between the parameters. However, since high correlations and low SMD are two conditions necessary and related to high levels of agreement, our study was able to provide a proxy of agreement. Nevertheless, a future study exploring the topic of agreement is required.

The current results have wide practical implications. Development of affordable and unconventional field test strategies, such as methods using heart rate variability [58], perceived exertion [59], and respiratory frequency [60], could allow the identification of the AerT with relatively good accuracy, simplicity, and velocity. From a physiological perspective, AerT can be considered as a “biomarker” for FAT_max_ identification (vice versa), which in turn could replace costly tests aiming to detect mentioned indices. From a practical point of view, the existence of a FAT_max_ and AerT association has important implications for both athletes and clinical populations, providing a basis for more applied and individualized training prescriptions since FAT_max_ training is defined as an effective method to enhance physical activity and fat oxidation [6,7]. This knowledge can improve training effectiveness by helping to recognize each subject’s unique physiological and energetic demands, allowing an accurate and highly tailored aerobic training prescription.

## 5. Conclusions

Several recommendations could be drawn from our results. First, the existence of methodological and clinical differences between and within the studies can play a decisive role when establishing the correlation between FAT_max_ and AerT. Using mL/min/kg as a measurement unit, short GXT stages, and gas analysis are recommended for standardization of future trials. In addition, the assessment should preferably be performed during one GXT session, whereas clinical differences, ergometer type, and FAT_max_ detection method, all have a trivial influence on correlation strength. Likewise, our MA showed non-homogenous PI results ranging from almost 0 to 1. Therefore, when non-standardized, *r* of the future studies is expected to range from almost no correlation to absolute correlation. Hence, we concluded that when methodological differences are standardized, a strong correlation between FAT_max_ and AerT is present.

## Figures and Tables

**Table 1:** Descriptive statistics of included studies with identified categorical moderator variables, categorised by differences in characteristics of the studies (methodological diversity) and study populations (clinical diversity).

Study ID	*N*		*r*	Participants Characteristics	Physical Activity Level	Ergometer	Exercise Intensity of FAT_max_	Exercise Intensity of AerT	Correlation Made Using	AeT Detection Method	VO_2max_ Protocol	FAT_max_ Detection Method	FAT_max_ Protocol
Achten et al., (2004) [49]	a	33	0.69	Male	Active	Cycle	73.4 ± 8.0%HR_max_	N/N	b/min	Lactate	Short	Visual	Identical as VO_2max_
	b		0.65	Male	Active	Cycle	63 ± 8.6%VO_2max_	60.9 ± 5.2%VO_2max_	L/min	Lactate	Short	Visual	Identical as VO_2max_
Bircher et al., (2004) [17]	a	10	0.75	Male	Active	Cycle	75%VO_2max_	77.29 ± 0.09%VO_2max_	L/min	Lactate	Short	Visual	Additional
	b		0.32	Male	Inactive	Cycle	65%VO_2max_	49.45 ± 0.11%VO_2max_	L/min	Lactate	Short	Visual	Additional
	c	10	0.67	Female	Active	Cycle	75%VO_2max_	76.44 ± 0.08%VO_2max_	L/min	Lactate	Short	Visual	Additional
	d		0.43	Female	Inactive	Cycle	65%VO_2max_	49.74 ± 0.12%VO_2max_	L/min	Lactate	Short	Visual	Additional
Bircher et al., (2005) [20]	a	48	0.56	Male	Active	Cycle	22.47 ± 8.01 mL/kg/min	27.73 ± 11.00 mL/min/kg	mL/min/kg	Lactate	Short	Visual	Identical as VO_2max_
	b		0.43	Male	Active	Cycle	106 ± 21 b/min	118 ± 30 b/min	b/min	Lactate	Short	Visual	Identical as VO_2max_
	c		0.37	Male	Active	Cycle	135 ± 26 b/min	127 ± 23 b/min	b/min	Lactate	Long	Visual	Identical as VO_2max_
	d		0.43	Male	Active	Cycle	32.5 ± 11.84 mL/kg/min	27.39 ± 12.56 mL/min/kg	mL/min/kg	Lactate	Long	Visual	Identical as VO_2max_
	e	30	0.57	Female	Active	Cycle	21.57 ± 6.09 mL/kg/min	25.76 ± 8.99 mL/min/kg	mL/min/kg	Lactate	Short	Visual	Identical as VO_2max_
	f		0.39	Female	Active	Cycle	113 ± 19 b/min	123.9 ± 20.3 b/min	b/min	Lactate	Short	Visual	Identical as VO_2max_
	g		0.46	Female	Active	Cycle	140 ± 23 b/min	125 ± 18.7 b/min	b/min	Lactate	Long	Visual	Identical as VO_2max_
	h		0.73	Female	Active	Cycle	31.36 ± 9.28 mL/kg/min	26.32 ± 10.00 mL/min/kg	mL/min/kg	Lactate	Long	Visual	Identical as VO_2max_
Emerenziani et al., (2019) [18]	a	52	0.94	Female	Inactive	Treadmill	121.6 ± 17.33 b/min	123.3 ± 16 b/min	b/min	Gas analysis	Short	Mathematical	Identical as VO_2max_
	b		0.82	Female	Inactive	Treadmill	14.7 ± 2.37 mL/kg/min	15.13 ± 2.17 mL/min/kg	mL/min/kg	Gas analysis	Short	Mathematical	Identical as VO_2max_
	c		0.93	Female	Inactive	Treadmill	69.13 ± 9.07%HR_max_	68.87 ± 8.87%HR_max_	%HR_max_	Gas analysis	Short	Mathematical	Identical as VO_2max_
	d		0.76	Female	Inactive	Treadmill	70.43 ± 11.43%VO_2max_	72.87 ± 10.4%VO_2max_	%VO_2max_	Gas analysis	Short	Mathematical	Identical as VO_2max_
Gmada et al., (2013) [51]	a	12	0.85	Male	Inactive	Cycle	N/N	N/N	%VO_2max_	Gas analysis	Short	Mathematical	Additional
Gonzalez-Haro (2011) [21]	a	11	−0.02	Male	Active	Cycle	52.3 ± 7.2%VO_2max_	67.3 ± 12.9%VO_2max_	%VO_2max_	Lactate	Long	Mathematical	Identical as VO_2max_
	b	11	0.37	Male	Active	Cycle	52 ± 5.7%VO_2max_	63.7 ± 12.9%VO_2max_	%VO_2max_	Lactate	Long	Mathematical	Identical as VO_2max_
Meucci et al., (2016) [52]	a	50	0.87	Male	Active	Treadmill	47.4 ± 4.25%VO_2max_	45.54 ± 4.07%VO_2max_	%VO_2max_	Gas analysis	Short	Visual	Identical as VO_2max_
Michallet et al., (2008) [19]	a	9	0.95	Male	Active	Cycle	1.4 ± 0.4 L/min	1.7 ± 0.6 L/min	L/min	Gas analysis	Short	Mathematical	Additional
	b	5	0.52	Female	Active	Cycle	1.4 ± 0.4 L/min	1.6 ± 0.4 L/min	L/min	Lactate	Short	Mathematical	Additional
Nikolovski et al., (2021) [12]	a	22	0.80	Male	Active	Cycle	21.34 ± 3.64 mL/min/kg	22.15 ± 4.84 mL/min/kg	mL/min/kg	Gas analysis	Short	Mathematical	Identical as VO_2max_
	b		0.73	Male	Active	Cycle	61.52 ± 7.24%HR_max_	63.38 ± 9.75%HR_max_	%HR_max_	Gas analysis	Short	Mathematical	Identical as VO_2max_
Peric et al., (2018) [22]	a	57	0.88	Male	Active	Treadmill	24.74 ± 5.52 mL/min/kg	23.95 ± 5.32 mL/min/kg	mL/min/kg	Gas analysis	Short	Visual	Identical as VO_2max_
Peric et al., (2020) [53]	a	19	0.89	Male	Inactive	Treadmill	17.23 ± 1.02 mL/kg/min	17.32 ± 1.10 mL/min/kg	mL/min/kg	Gas analysis	Short	Visual	Identical as VO_2max_
Rynders et al., (2011) [54]	a	74	0.77	Male	Inactive	Cycle	14.0 ± 7.3 mL/min/kg	15.4 ± 5.7 mL/min/kg	mL/min/kg	Lactate	Short	Visual	Identical as VO_2max_
	b	74	0.85	Female	Inactive	Cycle	11.4 ± 7.5 mL/min/kg	12.7 ± 5.8 mL/min/kg	mL/min/kg	Lactate	Short	Visual	Identical as VO_2max_
Venables et al., (2005) [55]	a	157	0.20	Male	Inactive	Treadmill	45 ± 12.53%VO_2max_	63 ± 12.53%VO_2max_	%VO_2max_	Gas analysis	Short	Visual	Identical as VO_2max_
	b	143	0.23	Female	Inactive	Treadmill	52 ± 11.96%VO_2max_	67 ± 11.96%VO_2max_	%VO_2max_	Gas analysis	Short	Visual	Identical as VO_2max_
Bircher et al., (2005) [50]	a	15	0.495	Female	Inactive	Cycle	65%VO_2max_	52.08 ± 12.13%VO_2max_	mL/min/kg	Lactate	Short	Visual	Additional
	b		0.655	Female	Inactive	Cycle	65%VO_2max_	50.18 ± 7.14%VO_2max_	mL/min/kg	Gas analysis	Short	Visual	Additional
	c	13	0.377	Male	Inactive	Cycle	65%VO_2max_	46.83 ± 6.99%VO_2max_	mL/min/kg	Gas analysis	Short	Visual	Additional

*NOTE*: N/N intensity not reported, short (≤3-min) and long (>3-min), identical as VO_2max_ (FAT_max_ and AerT determined during one test) and additional (FAT_max_ determined during additional test with stages length > 3 min), different ES’s within the included studies have been marked with letters for identification and analysis purposes.

**Table 2:** Descriptive statistics of pooled ESs according to measurement units.

Measurement Unit	ES (*r*)	CI 95%	PI	I^2^ Level 2 (%)	I^2^ Level 3 (%)	Q	*p*
Beats per minute (b/min)	0.622	0.165 to 0.859	−0.600 to 0.973	90.46	-	62.4	<0.001
Percentage of maximal oxygen uptake (%VO_2max_)	0.561	0.124 to 0.816	−0.603 to 0.962	92.65	-	74.8	<0.001
Litres per minute (L/min)	0.686	0.385 to 0.863	−0.207 to 0.955	60.95	-	14.7	0.023
Millilitres per kilogram per minute (mL/kg/min)	0.777	0.642 to 0.865	0.320 to 0.941	5.36	69.9	45.7	<0.001

*NOTE*: %HR_max_ failed to fulfill the inclusion criteria for statistical significance and to accurately estimate heterogeneity.

**Table 3:** Subgroups meta-analysis for SMD. *NOTE*: A negative mean indicates FAT_max_ preceded AerT.

Moderator and Groups	SMD				Heterogeneity Test		I^2^	
	Mean	SE	(95% CI)	[95% PI]	Q Statistic	*p*	2-Level (%)	3-Level (%)
Sex								
Males	−0.17	0.19	(−0.58, 0.25)	[−1.49, 1.16]	Q_13_ = 187.1	<0.001	40.8	51.9
Females	−0.20	0.17	(−0.59, 0.18)	[−1.39, 0.98]	Q_9_ = 103.5	<0.001	89.4	2.4
**Physical activity level**								
Active	−0.12	0.21	(−0.56, 0.33)	[−1.49, 1.25]	Q_16_ = 131.4	<0.001	53.4	37.8
Inactive	−0.45	0.22	(−0.96, 0.05)	[−1.55, 0.64]	Q_8_ = 92.1	<0.001	1.3	90.1
**Ergometer**								
Treadmill	−0.07	0.32	(−0.81, 0.67)	[−1.86, 1.72]	Q_8_ = 184.3	<0.001	1.7	94.8
Cycling	−0.29	0.17	(−0.64, 0.06)	[−1.38, 0.81]	Q_16_ = 101.0	<0.001	65.4	22.6
**VO_2max_ test**								
Long stages	0.09	0.29	(−0.65, 0.82)	[−1.75, 1.93]	Q_5_ = 30.1	<0.001	91.2	/
Short stages	−0.32	0.11	(−0.54, −0.09)	[−1.27, 0.64]	Q_19_ = 207.1	<0.001	89.4	/
**AerT detection method**								
Gas analysis	−0.23	0.26	(−0.80, 0.35)	[−1.80, 1.35]	Q_24_ = 189.5	<0.001	1.3	93.9
Lactate analysis	−0.22	0.18	(−0.62, 0.17)	[−1.38, 0.93]	Q_13_ = 92.03	<0.001	71.6	17.8
**FAT_max_ detection method**								
Visual	−0.07	0.23	(−0.56, 0.41)	[−1.52, 1.37]	Q_15_ = 281.4	<0.001	46.7	48.3
Mathematical	−0.48	0.17	(−0.86, −0.09)	[−1.26, 0.31]	Q_9_ = 19.4	0.022	8.6	62.2

## Data Availability

Not applicable.
